# Nature-inspired nanocarriers for improving drug therapy of atherosclerosis

**DOI:** 10.1093/rb/rbad069

**Published:** 2023-08-11

**Authors:** Weihong Ji, Yuanxing Zhang, Yuanru Deng, Changyong Li, Ranjith Kumar Kankala, Aizheng Chen

**Affiliations:** Institute of Biomaterials and Tissue Engineering, Huaqiao University, Xiamen, Fujian 361021, PR China; Fujian Provincial Key Laboratory of Biochemical Technology, Huaqiao University, Xiamen, Fujian 361021, PR China; The Institute of Forensic Science, Xiamen Public Security Bureau, Xiamen, Fujian 361104, PR China; Institute of Biomaterials and Tissue Engineering, Huaqiao University, Xiamen, Fujian 361021, PR China; Fujian Provincial Key Laboratory of Biochemical Technology, Huaqiao University, Xiamen, Fujian 361021, PR China; Institute of Biomaterials and Tissue Engineering, Huaqiao University, Xiamen, Fujian 361021, PR China; Fujian Provincial Key Laboratory of Biochemical Technology, Huaqiao University, Xiamen, Fujian 361021, PR China; Institute of Biomaterials and Tissue Engineering, Huaqiao University, Xiamen, Fujian 361021, PR China; Fujian Provincial Key Laboratory of Biochemical Technology, Huaqiao University, Xiamen, Fujian 361021, PR China; Institute of Biomaterials and Tissue Engineering, Huaqiao University, Xiamen, Fujian 361021, PR China; Fujian Provincial Key Laboratory of Biochemical Technology, Huaqiao University, Xiamen, Fujian 361021, PR China

**Keywords:** nature-inspired nanocarriers, drug delivery, membrane-coating, stimuli-responsive, atherosclerosis

## Abstract

Atherosclerosis (AS) has emerged as one of the prevalent arterial vascular diseases characterized by plaque and inflammation, primarily causing disability and mortality globally. Drug therapy remains the main treatment for AS. However, a series of obstacles hinder effective drug delivery. Nature, from natural micro-/nano-structural biological particles like natural cells and extracellular vesicles to the distinctions between the normal and pathological microenvironment, offers compelling solutions for efficient drug delivery. Nature-inspired nanocarriers of synthetic stimulus-responsive materials and natural components, such as lipids, proteins and membrane structures, have emerged as promising candidates for fulfilling drug delivery needs. These nanocarriers offer several advantages, including prolonged blood circulation, targeted plaque delivery, targeted specific cells delivery and controlled drug release at the action site. In this review, we discuss the nature-inspired nanocarriers which leverage the natural properties of cells or the microenvironment to improve atherosclerotic drug therapy. Finally, we provide an overview of the challenges and opportunities of applying these innovative nature-inspired nanocarriers.

## Graphical abstract

**Figure:**
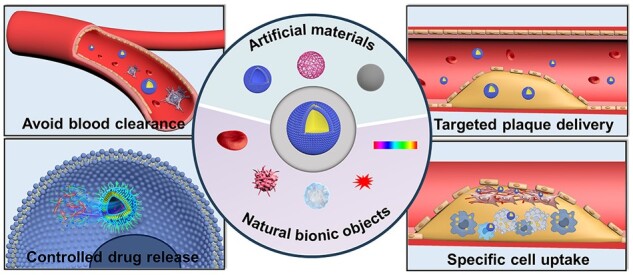

## Introduction

Atherosclerosis (AS) is the primary cause of cardiovascular and cerebrovascular diseases (CVDs) [1]. In 2020, the total number of CVD-associated deaths worldwide reached 20 million, accounting for 44% of all deaths from non-communicable diseases [2, 3]. AS is the most common arterial vascular disease, usually accompanied by abnormal lipid metabolism, plaque generation and inflammation [4]. The main risk factor for AS is elevated low-density lipid proteins (LDL) in the blood [5]. The onset of the disease begins in the intima of the artery with lipid accumulation, followed by fibrous tissue hyperplasia and calcification. With the proliferation of smooth muscle cells and the invasion of immune cells, the blood vessel walls swell and form atherosclerotic plaques [6]. The atherosclerotic plaques severely impede the flow of oxygen-rich blood to the heart, brain and other organs, which is the pathological basis of CVDs, such as coronary artery disease, stroke and peripheral artery disease [7, 8].

Currently, the clinical treatment for AS includes drug therapy and surgery [9, 10]. Drug therapy mainly relies on statins for lipid regulation combined with aspirin, clopidogrel or other drugs for anti-platelet therapy to prevent the development of obstructive vascular lesions [11, 12]. However, statins often require long-term or lifelong use, causing serious side effects, such as gastrointestinal reactions, liver damage and muscle damage [13, 14]. One of the main reasons for these side effects is their low selectivity of action. Free drugs usually exhibit weak affinity for the target tissue, leading to inadequate concentration at the lesion site and distribution in other normal tissues, thus affecting the efficacy and causing side effects. In addition to drug therapy, surgical interventions, such as stent implantation and vascular bypass, are mainly employed for patients with advanced and severe AS [15]. However, surgical interventions may cause side effects, including vascular restenosis and thrombosis, hampering the long-term efficacy of surgical intervention [16].

With the rapid development of nanotechnology, the research and development of nanomedicines have emerged as a key direction for drug innovation [17]. In recent years, the application of nanotechnology in disease diagnosis, treatment and monitoring has expanded significantly [2]. Industries related to nanomedicine are developing rapidly, with the constant emergence of new technologies, applications and achievements [18]. Compared to conventional pharmaceutical preparations, nanomedicines have specialized ultrasmall-sized nanostructures with active surface properties, which are the basis for enhancing the therapeutic effect of drugs [18]. Nanocarriers can satisfy therapeutic needs by overcoming various challenges in free drug delivery [19]. In terms of drug properties, nanocarriers can increase the solubility and stability of insoluble drugs, and improve drug dissolution or release behavior, thereby improving the bioavailability of drugs [20]. In addition, nanocarriers can enhance the selectivity of drugs toward specific tissues, organs or cells, realizing novel drug delivery routes of administration after special preparations [21]. With a reasonable design, nanocarriers can substantially meet therapeutic requirements, showing great application potential in various disease fields, such as severe tissue trauma, bacterial and virus infections, and malignant tumors [22–25].

Various common types of nanomaterials include liposomes, micelles, dendritic polymers and inorganic nanoparticles [26]. Most of the commercially available nanomedicines are based on liposomes [27]. For instance, Doxil (PEGylated liposomal doxorubicin) was the first Food and Drug Administration (FDA)-approved long-circulating liposome for treating tumors. In addition, Onivyde (PEGylated liposomal irinotecan) prevented the large-scale conversion of irinotecan to its carboxylate form under physiological conditions, resulting in significantly improved efficacy and reduced adverse reactions [28]. In recent years, nucleic acid drugs have developed rapidly with the breakthrough of key technologies such as delivery systems and chemical modifications [27, 29]. In 2018, the first siRNA drug Patisiran was approved by the FDA for treating patients with polyneuropathy caused by hereditary transthyretin-mediated amyloidosis (hATTR) [30]. Patisiran was fabricated using lipid nanoparticles (LNPs) to enclose and deliver siRNA. After intravenous administration, the LNPs ensure that they are not quickly filtered out by the kidney owing to the size effect. Instead, they can passively target liver tissue, facilitating the gradual uptake of siRNA by target cells. This mechanism plays a key role for Patisiran to overcome major constraints and getting market approval. In addition, the Moderna COVID-19 vaccine (mRNA-1273) is an mRNA vaccine encapsulated in LNPs [31]. LNPs are important components of the COVID-19 mRNA vaccine in protecting and transporting mRNA to cells. Although the field of nanomedicine is developing rapidly, there remains room for improvement in its effectiveness due to the unclear interaction mechanism between nanocarriers and organisms [32]. The interaction approaches between natural cells and the human body open new avenues for designing more efficient nanocarriers [33].

This article first systematically introduced and summarized the nanoparticle delivery barriers for AS treatment, including blood clearance, non-plaque target, nonspecific cellular uptake, and uncontrolled release of drug. According to the delivery barriers, the nature-inspired nanoparticles, including cell-inspired and microenvironment-responsive nanoparticles, were summarized and classified. It is worth mentioning that we highlighted the biomimetic features of nature-inspired nanocarriers for overcoming the delivery barriers, instead of focusing on the function of the drug core for complex pathologies. Finally, the challenges and perspectives of nature-inspired nanocarriers in clinical translation were analyzed and discussed.

## Materials and methods

A literature search was performed in PubMed and Web of Science databases without date limitation using the following terms: ‘atherosclerosis’ AND ‘nanoparticles’ AND ‘red blood cell’ OR ‘platelet’ OR ‘macrophage’ OR ‘exosome’ OR ‘microenvironment-responsive’. The language was restricted to English.

## Delivery barriers for as treatment

**Figure 1.**
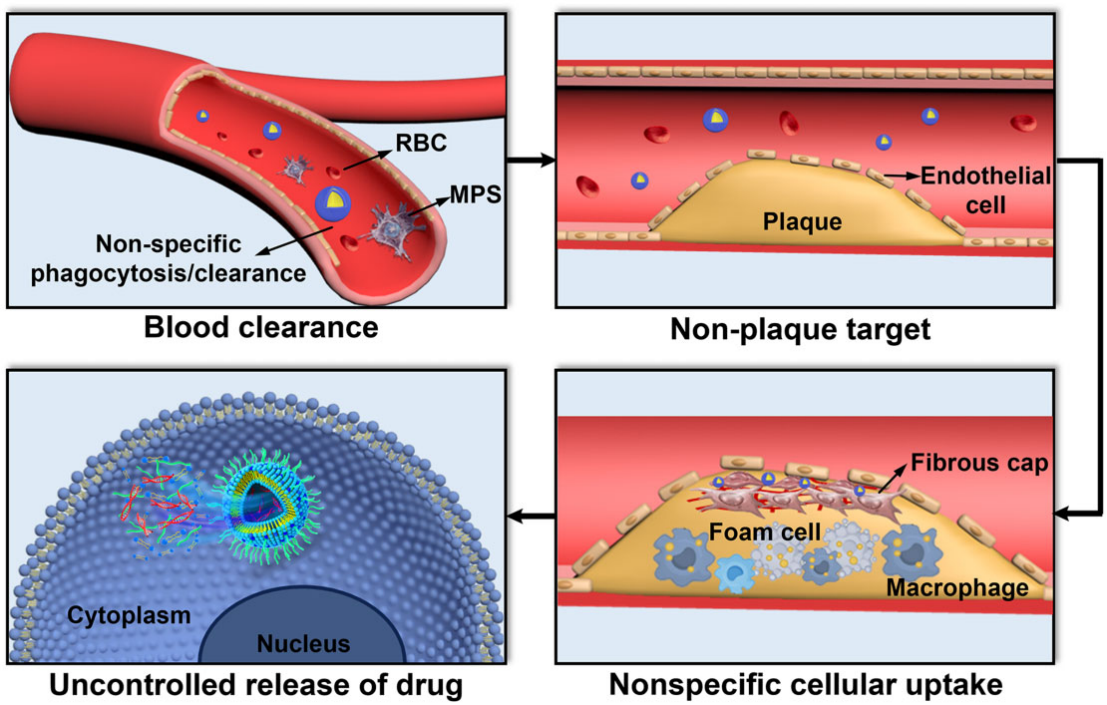
The delivery challenges in nanomedicines for as treatment.

Nanomaterials provide powerful drug delivery platforms for delivering drugs to treat AS [34]. Flores *et al.* systematically summarized targeted nanoparticle therapy for AS different pathologies [34]. However, there exist several delivery challenges of nanomedicine for AS treatment (Figure 1). Firstly, the therapeutic efficacy of nanomedicines is greatly reduced by the non-specific clearance due to the mononuclear phagocyte system (MPS) [35]. The MPS consists of a series of phagocytosis cells that can mediate blood circulation time and biodistribution of nanocarriers. These cells can rapidly clear nanocarriers, resulting in inadequate concentrations of nanocarriers in the blood [36]. AS is a vascular disease, and the low concentration of drugs in the blood vessels seriously affects the therapeutic effect of drugs at the plaque site (Figure 1). As mentioned above, the incorporation of PEG in nanocarriers can prolong blood circulation time to some extent and improve the efficacy of drugs [37]. However, an increasing number of studies have shown that repeated injections of PEG can trigger an immune response, ultimately leading to accelerated blood clearance [38, 39]. Natural cells, such as red blood cells (RBCs), have a lifespan of 120 days which is an unattainable long-acting time for almost all drugs and nanocarriers [40]. Therefore, RBC membranes (RBCm) are often used to carry drugs to achieve long circulation [41].

Over the past few decades, atherosclerotic cardiovascular mortality has been reduced by lowering LDL cholesterol or addressing other traditional risk factors, such as diet, lifestyle, obesity and hypertension [12]. However, the overall benefits of therapeutic strategies for these risks have stagnated, and the global health and economic burden of atherosclerotic cardiovascular disease remains substantial. Targeting treatment toward plaques can effectively alleviate the bottleneck faced by current treatment approaches [42]. Previous studies reported that plaque development was accompanied by inflammation [7, 43]. Considering the relationship between atherosclerotic plaque and inflammation, nanomaterials with inflammation-targeting function could realize drug enrichment in plaques (Figure 1) [8]. Immune cells possess the natural tendency to inflammatory tissues or sites, such as leukocytes and macrophages [44, 45]. Considerably, the membrane coating can confer the corresponding function of the source cell to the drug carrier, thereby achieving plaque-targeted delivery [46, 47].

Pathological macrophages and foam cells play an important role in promoting early and advanced atherosclerotic lesions [48, 49]. In early atherosclerotic lesions, macrophages engulf excess lipoproteins and form foam cells within the intima [50]. As inflammation progresses, macrophages suffer massive apoptosis and cannot be cleared in time, triggering the formation and development of vulnerable plaques [51, 52]. Therefore, macrophages are the main target for AS treatment. However, nanocarriers must penetrate through plaque with a thick fibrous cap before reaching the pathological macrophages [53]. In addition, the complex plaque microenvironment makes it difficult for nanocarriers to target dysfunctional cells (Figure 1). In atherosclerotic plaques, there are complex cellular components and high levels of reactive oxygen species (ROS) [54, 55]. Stimuli-responsive materials have been used to construct drug delivery systems with flexible structures, enriching drugs at action sites [2].

## Construction strategies

**Figure 2.**
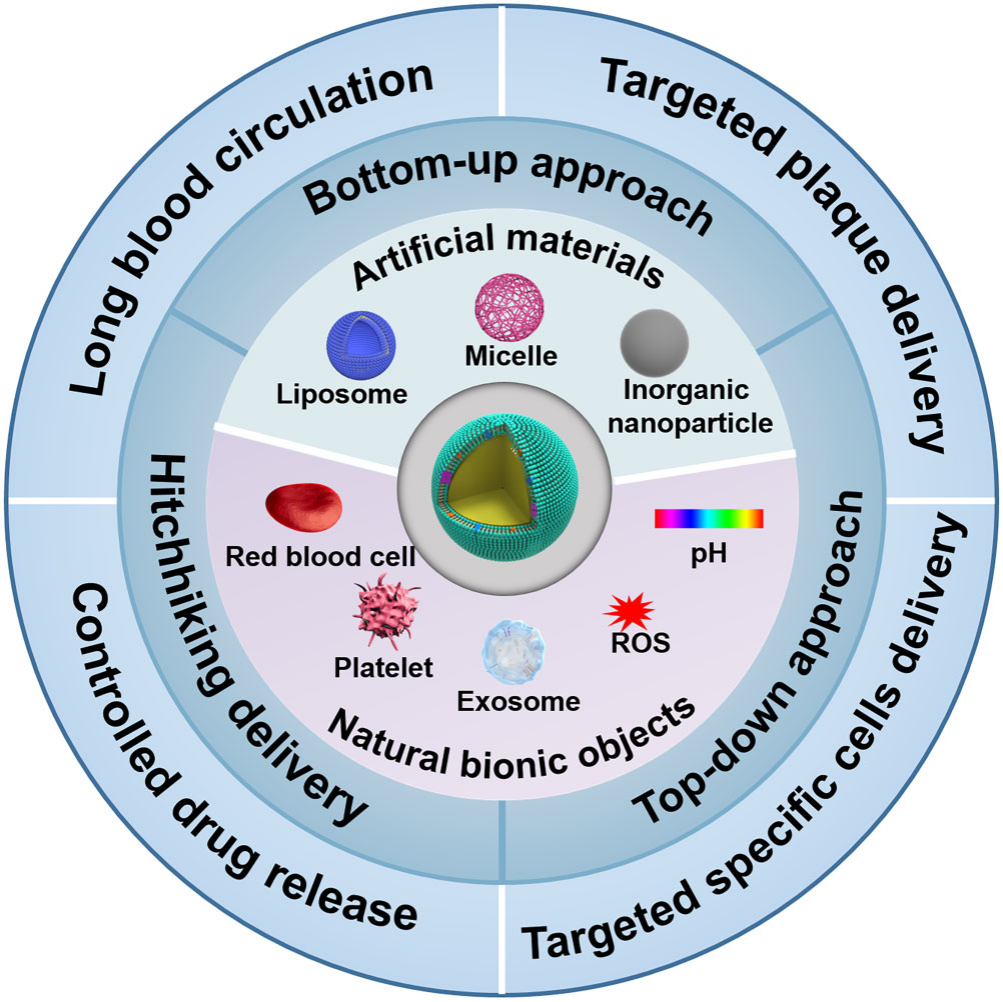
A schematic overview of nature-inspired nanocarriers for drug delivery to treat as.

Broadly speaking, there exist three types of approaches to construct nature-inspired nanocarriers (Figure 2). The first approach is the bottom-up approach [44, 56]. The bottom-up approach is one of the important approaches in nanotechnology and nanomanufacturing. The approach generally refers to the strategy of starting with individual molecules or simple components, and then building more complex structures [57]. To simulate the function of the natural bionic objects as much as possible, the bottom-up approach typically utilizes functional components of objects, such as lipid molecules and proteins, for constructing biomimetic nanocarriers through simple incubation, chemical bonding and other techniques [57, 58]. For instance, exosomes (Exos) have been developed for the delivery of pharmaceutical proteins and nucleic acids [58]. However, not all components in natural Exos are required for efficient delivery and the considerable complexity of Exos might affect their clinical transformation. A viable alternative is to assemble synthetic Exo mimetics by lipid molecules and functional proteins [59]. This approach allows a controlled preparation process to generate well-characterized biomimetic nanocarriers. However, it is not sufficient to fully replicate the complexity or functionality of bionic objects. In contrast to the bottom-up approach, the top-down approach refers to the strategy of starting from complex substrates, and then crushing into small and simple units for the assembly of nanostructures [58]. The top-down approach can replicate the necessary lipids and proteins of bionic objects in one step, imparting biocompatibility and corresponding functions to nanocarriers [45, 60, 61]. Membrane-coating provides an effective top-down strategy that reconstructs micrometer-scale cell membranes into nanovesicle structures with source cell functions [62–65]. For example, RBC membrane has been widely used to constitute biologic or hybrid nanocarriers capable of greatly enhancing pharmacokinetics [41]. However, the biophysics involved in the membrane coating process is not satisfactorily understood, requiring the improvement of the manufacturing method. The third method is to modify cells with drugs or drug carriers and realize targeted drug delivery in the hitchhiking delivery system [66–70]. This method utilizes the self-homing ability of cells to achieve drug enrichment in the action site.

In conclusion, natural cells and extracellular vesicles (EVs), including RBCs, platelets, white blood cells, macrophages and exosomes, can be combined with synthetic materials for efficient drug delivery. On the one hand, synthetic materials can enable the loading of drugs with different properties, such as hydrophobic chemical drugs and easily degradable and negatively charged nucleic acid drugs. Some synthetic nanomaterials can exploit their microenvironmental reactivity or endosomal escape properties [71, 72]. On the other hand, membrane-coated nanocarriers can effectively interact with surrounding proteins, cells and other biological substrates after administration *in vivo*. Bionic design can give nanocarrier characteristics, such as self-marking, natural tropism and cell entry mechanisms, to enhance the therapeutic effect of drugs. Therefore, the nature-inspired nanocarriers with optimized natural properties are expected to overcome drug delivery barriers for AS treatment (Figure 2).

## Nature-inspired nanocarriers

Micro-/nano-structural biological particles, such as cells and EVs, have been widely used for constructing drug carriers [58, 73, 74]. Table 1 lists nanocarriers inspired by natural cells and EVs, along with their corresponding construction strategies. Owing to the functional components of biomimetic objects, these nature-inspired nanocarriers can overcome delivery barriers and efficiently deliver drugs for AS treatment.

**Table 1.** Summary of nanocarriers inspired by natural cells and vesicles

Biomimetic objects	Strategies	Nanocarriers	Functional moieties	Delivery effects	References
Red blood cell	RBCm-coating	RBC/RAP@PLGA	CD47	Long blood circulation	[75]
	CR8-modified phospholipid hybrid RBCm	RBC@DTX/CR8	CD47/targeting peptide CR8	Long blood circulation/targeted endothelial cell delivery	[76]
Platelet	Platelet membrane-coating	PM-PAAO-UCNPs	P-selection	Targeted plaque delivery	[77]
	Platelets-liposomes hybrid	P-Lipo	P-selection/CD 40L/ICAM-2	Targeted plaque delivery	[78]
	Platelet-like fusogenic liposomes	PLM-miRs	P-selection	Targeted macrophage delivery	[79]
Macrophage	Macrophage membrane-coating	MM/RAPNP	CD47/Integrin α4/Integrin β1	Long blood circulation/targeted plaque delivery	[80]
	Macrophage-RBCm hybrid coating	HA-M@PB@ (PC + ART)	CD47/CD11b	Long blood circulation/targeted macrophage delivery	[81]
	Macrophage-hitchhiking delivery	HA-Fc/${\text{NP}}_{\text{ST}}^{3}$	β-cyclodextrin (β-CD)/adamantane	Targeted plaque delivery	[82]
Extracellular vesicles (EVs)	Platelet-derived EVs	MCC950-PEVs	P-selection	Targeted plaque delivery	[83]
	Platelet membrane modified MSCs-EVs	P-EVs	GPVI/Integrin α2β1/CD62P	Targeted plaque delivery	[84]
	Exosome-mediated delivery	Exo^IRES-^^*IL-10*^	TSG101/CD9	Efficient macrophage delivery	[85]
		M2 macrophages-derived exosomes HAL@M2 Exo	Chemokine receptors	Targeted inflammation delivery	[86]

### RBCs-inspired nanocarriers

**Figure 3.**
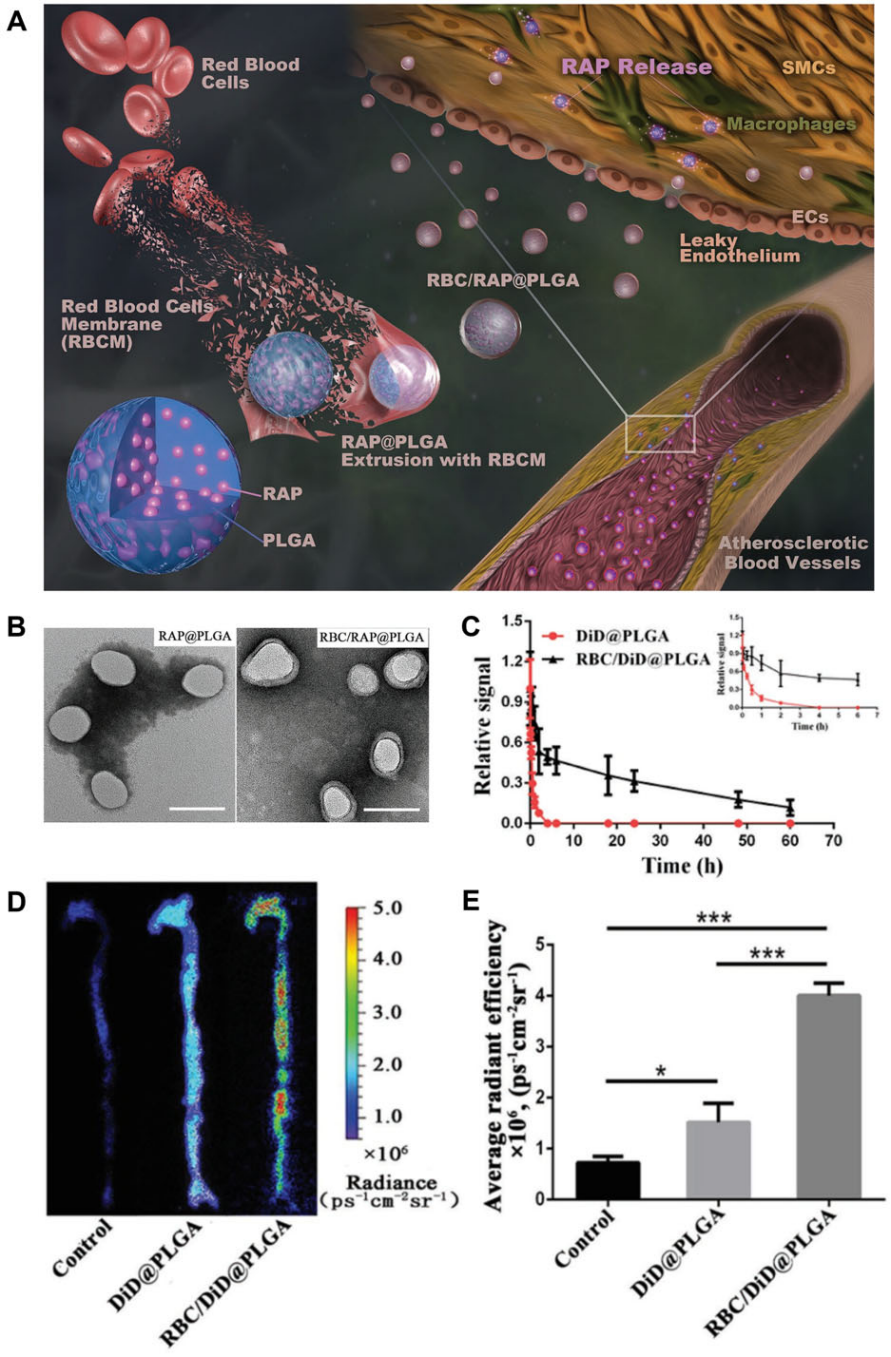
RBCm-coated nanoparticles RBC/RAP@PLGA for treatment of as. (**A**) Schematic illustration of RBC/RAP@PLGA for as treatment. (**B**) Transmission electron microscope images of nanoparticles. Scale bar: 100 nm. (**C**) Pharmacokinetic studies of RBC/DiD@PLGA and DiD@PLGA. (**D**) The *ex vivo* fluorescence images of the aorta and (**E**) quantitative data of fluorescence signals accumulated in the aorta of ApoE^−^/^−^ mice treated with different formulations (*n* = 3). Adapted with permission from Ref. [75].

Translocating RBC membranes to the surface of synthetic nanoparticles can generate stealth carriers with extended circulation capabilities [87]. Wang *et al.* fabricated the RBC-based ‘core–shell’ structured nanocomplexes (Figure 3A) [75]. Specifically, the poly(lactic-*co*-glycolic) acid (PLGA) nanoparticles were loaded with rapamycin (RAP) as a ‘core’ structure (RAP@PLGA), and RBC vesicles extracted from RBCs were coated on the surface of RAP@PLGA to construct the RBC/RAP@PLGA composites (Figure 3B). The RBC/DiD@PLGA exhibited ∼60% and 31% overall retention in blood after 4 and 24 h injection, respectively (Figure 3C). However, the bare DiD@PLGA showed a neglected signal after 4 h injection, indicating rapid blood clearance. Compared with DiD@PLGA, a stronger fluorescence signal of RBC/DiD@PLGA accumulated in atherosclerotic plaque areas (Figure 3D and E). These results demonstrated that the RBC membrane coating strategy prolonged the half-time in circulation and enhanced atherosclerotic plaque targeting *in vivo*. Moreover, the modified erythrocyte membrane offers additional functions, such as improved targeting and controlled drug release [76, 88]. Zhong *et al.* prepared the targeted polypeptide CR_8_-modified phospholipid polymer DSPE-PEG2000-CR_8_ for constructing the hybrid erythrocyte platform. The drug docetaxel (DTX) was modified on the surface of the platform with an acid-cleavable ester bond [76]. Due to the advantages of CR_8_-mediated active targeting and pH-sensitive prodrugs, the functionalized erythrocyte platform achieved efficient and safe AS management.

### Platelets-inspired nanocarriers

**Figure 4.**
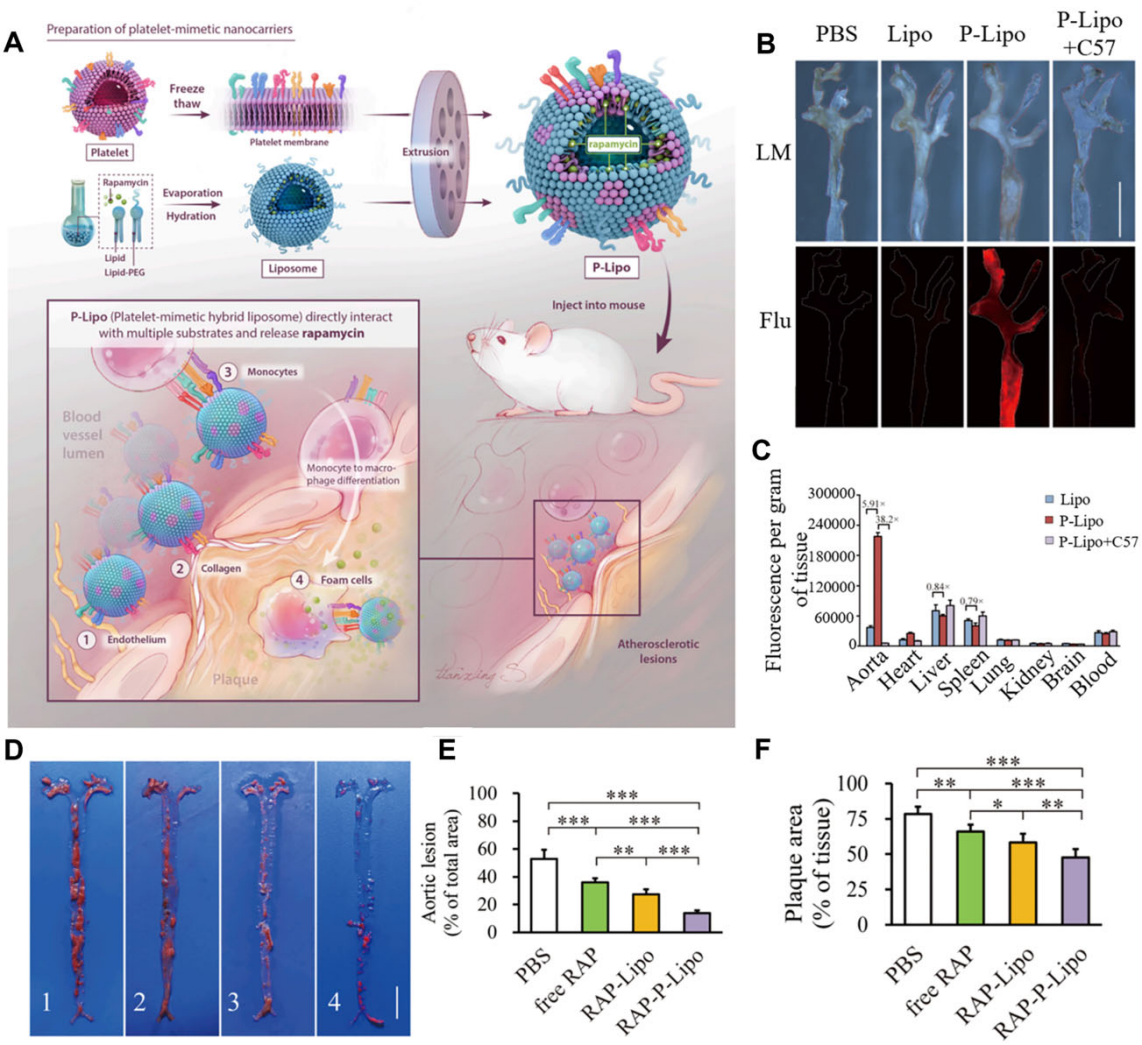
Platelet-mimetic nanocarriers for targeted therapy of AS. (**A**) Schematic illustration of P-Lipo preparation and its targeting treatment for AS. (**B**) White light images and fluorescence images (flu) of the aorta after injection of different formulations. Scale bar: 5 mm. (**C**) Biodistribution in major organs of mice 2 h after intravenous injection. (**D**) Representative images of total aortas from each group. Quantitative analysis of (**E**) aortic lesion and (**F**) plaque area. Adapted with permission from Ref. [78].

Platelets are involved in the pathological processes of many inflammatory diseases, including cancer, infection and AS [89]. Studies have shown that platelets could directly interact with inflammatory tissues and cells, such as damaged endothelial cells, monocytes and macrophages, during the initiation and progression of AS [90–92]. The P-selectin expressed on platelets could bind to P-selectin glycoprotein ligand-1 (PSGL-1) expressed by inflammatory cells [93, 94]. Additionally, other receptor–ligand pairs, such as LFA-1 and MAC-1 via platelet ICAM-2, and GP Ibα and CD40 via platelet CD40L have been demonstrated [90, 91]. Due to the homing ability of platelets in atherosclerotic plaque, platelet-inspired nanocarriers provide an efficient platform for targeted drug delivery to plaque [77, 78]. Song *et al.* designed biomimetic liposomes (P-Lipo) by hybridizing platelet membranes with artificial liposomes for atherosclerotic plaque targeting (Figure 4A) [78]. The P-Lipo directly interacted with multiple substrates and showed a 5.91-fold increase in fluorescent signal accumulation into atherosclerotic plaques (Figure 4B and C). Meanwhile, the accumulation of fluorescence intensity of P-Lipo in diseased aortas was significantly stronger than that in normal aortas and other organs (Figure 4C), indicating that P-Lipo possessed good plaque specificity and safety. P-Lipo significantly reduced the growth of atherosclerotic lesion from 52.92 ± 6.66% to 13.83 ± 2.09% and showed excellent anti-AS effects (Figure 4D–F).

For RNA drugs, in addition to targeted delivery, the low cytoplasmic drug concentration caused by lysosomes needs to be considered [29, 95]. Studies indicated that only 1–2% of RNA could be dispersed into the cytoplasm by lysosomes through endocytosis [96]. To increase the concentration of nucleic acid drugs in plaque target cells, Tan *et al.* constructed platelet-like fusogenic liposomes (PLPs) for loading and delivering the microRNA (miRNA) [79]. The PLPs carried miRNA to monocytes/macrophages in the injured area and directly released miRNA into the cytoplasm through membrane fusion, which enhanced the miRNA concentrations at the action sites and achieved efficient therapy.

### Macrophages-inspired nanocarriers

**Figure 5.**
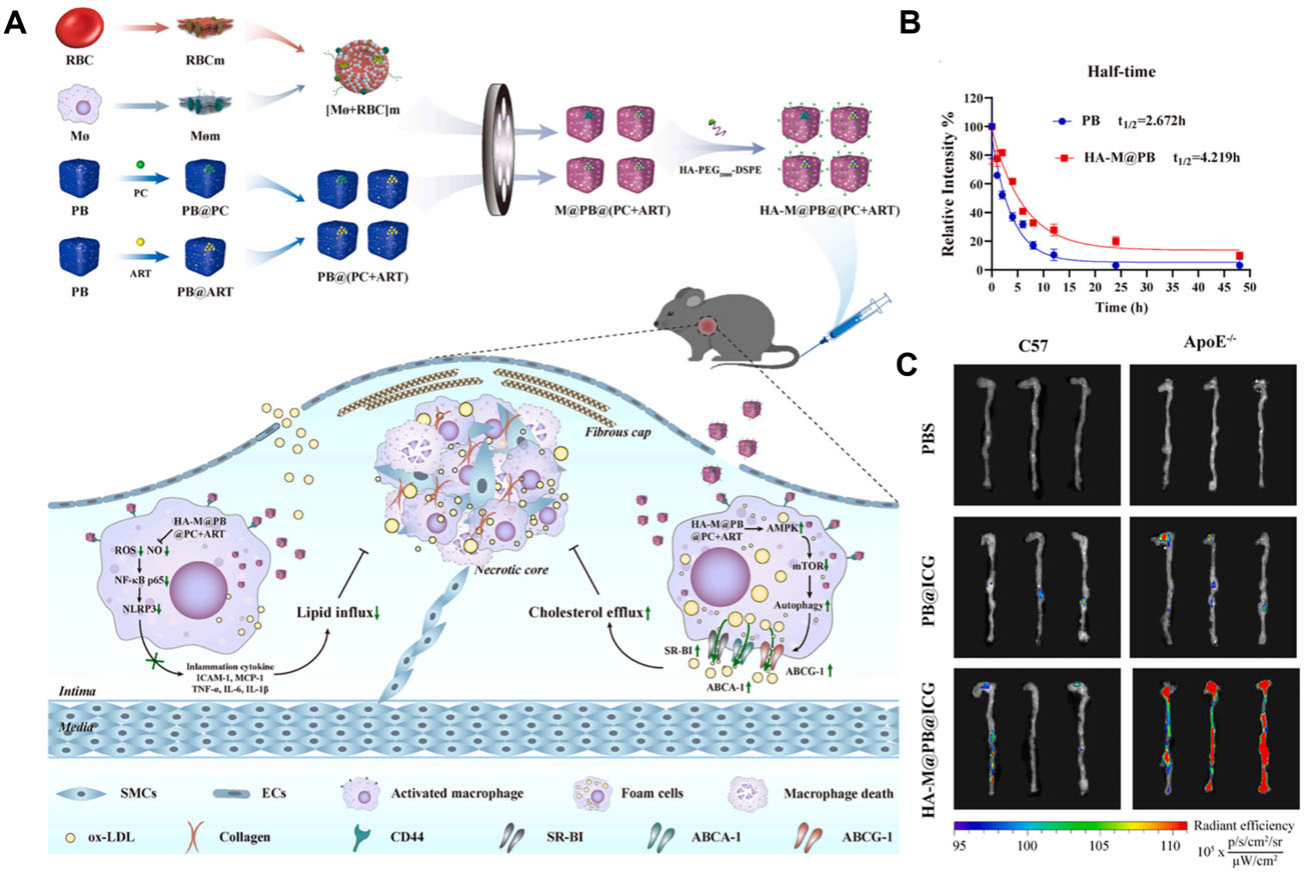
Biomimetic membrane-coated HA-M@PB@ (PC + ART) NPs for treatment of AS. (**A**) Schematic illustration of HA-M@PB@ (PC + ART) NPs and their treatment for AS. (**B**) *In vivo* pharmacokinetic curves after intravenous injection of PB and HA-M@PB. (**C**) The fluorescence images of the aorta after intravenous injection of different formulations. Adapted with permission from Ref. [81].

During the formation and development of atherosclerotic plaques, macrophages are recruited and migrated into the arterial vessel wall [49, 97]. Inspired by this aspect, macrophage membranes are frequently used to construct targeted delivery systems [47, 80, 98]. Zhou *et al*. prepared multifunctional nanocomplexes (HA-M@PB) by combining the long circulation lifetime of RBCs and the targeted capacity of macrophages (Figure 5A) [81]. Compared to Prussian blue nanoparticles (PB NPs) without membrane coating, the HA-M@PB NPs exhibited an extended blood retention time of 1.73 times (Figure 5B). As shown in Figure 5C, strong fluorescence signals were observed in the aortic arch and abdominal aorta of ApoE^−^/^−^ mice treated with HA-M@PB NPs, while PB NPs treated ApoE^−^/^−^ mice showed only weak fluorescence signals. These results demonstrated that the hybrid membrane of erythrocytes and macrophages endowed the nanocarriers with long circulation and targeting capabilities.

**Figure 6.**
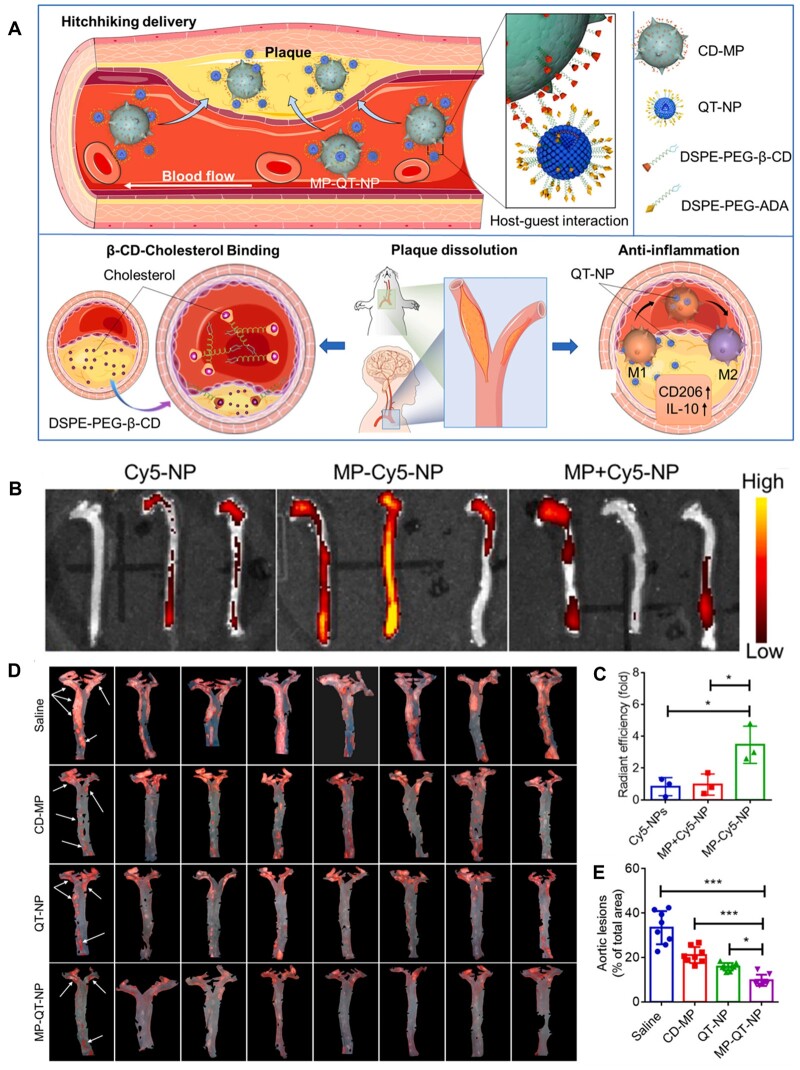
Macrophage-mediated hitchhiking delivery for treatment of AS. (**A**) Schematic illustration of hitchhiking delivery. (**B**) The fluorescence images of the aorta after intravenous injection and (**C**) quantitative analysis by using *in vivo* imaging system. (**D**) The microscope images of aortic lesions and (**E**) quantitative data of lesions on the intimal surface of the aorta. Adapted with permission from Ref. [82].

Considering the pathological characteristics of macrophage recruitment during plaque formation and development, macrophage hitchhiking delivery systems via host–guest interactions were constructed for targeted anti-AS therapy [99, 100]. A β-cyclodextrin (β-CD) derivative was used to modify the macrophage (CD-MP), and adamantane (ADA) was modified on the surface of quercetin (QT)-loaded liposome (QT-NP) [82]. By the host–guest interaction between β-CD and ADA, the drug-loaded liposomes were conjugated to CD-MP, forming the hitchhiking delivery system (MP–QT-NP) (Figure 6A). The ApoE^−^/^−^ mice treated with supramolecular conjugate MP–Cy5-NP showed strong fluorescence signals in the aorta, ∼3.9-fold than that of the Cy5-NP group (Figure 6B and C). The mice treated with a simple mixture of MP and Cy5-NP (MP + Cy5-NP) exhibited low fluorescence signals. These results indicated that the ‘hand-in-hand’ delivery between MP and NP significantly improved the concentration of drugs at focal sites, possessing a good targeting effect. MP–Cy5-NP effectively reduced the area of aortic lesion (% of total area) to 20.4% in comparison to 34.6% of the saline group (Figure 6D and E), which could be attributed to the β-CD-cholesterol binding and anti-inflammation of QT-NP after macrophage-hitchhiking targeted delivery to the atherosclerotic plaque. These findings suggested that the hitchhiking delivery strategy substantially relied on the self-homing ability of cells and the interaction between nanoparticles and cells.

### EVs-inspired nanocarriers

EVs are membranous vesicles ranging from 30 nm to 5 μm in size released by cells into the extracellular matrix, including exosomes, microvesicles and apoptotic bodies [101]. EVs can steadily carry important signaling molecules involved in cell communication, migration, angiogenesis and tumor cell growth [102]. The development of AS involves the participation of various cells, including endothelial cells, vascular smooth muscle cells, platelets and macrophages [103, 104]. Activation or apoptosis of these cells is accompanied by secretion and shedding of associated EVs. EVs from these cells can promote inflammation and plaque formation by mediating cell-to-cell communication. Due to the inflammatory targeting ability of platelets, platelet vesicles also exhibit an inflammatory tendency and can accumulate at the site of atherosclerotic plaques [83, 105]. Ma *et al*. engineered platelet-derived EVs loaded with the NLRP3-inflammasome inhibitor MCC950 (MCC950-PEVs) [83]. Compared to the free drug MCC950, MCC950-PEVs could target the damaged aorta and significantly reduce atherosclerotic plaque size.

**Figure 7.**
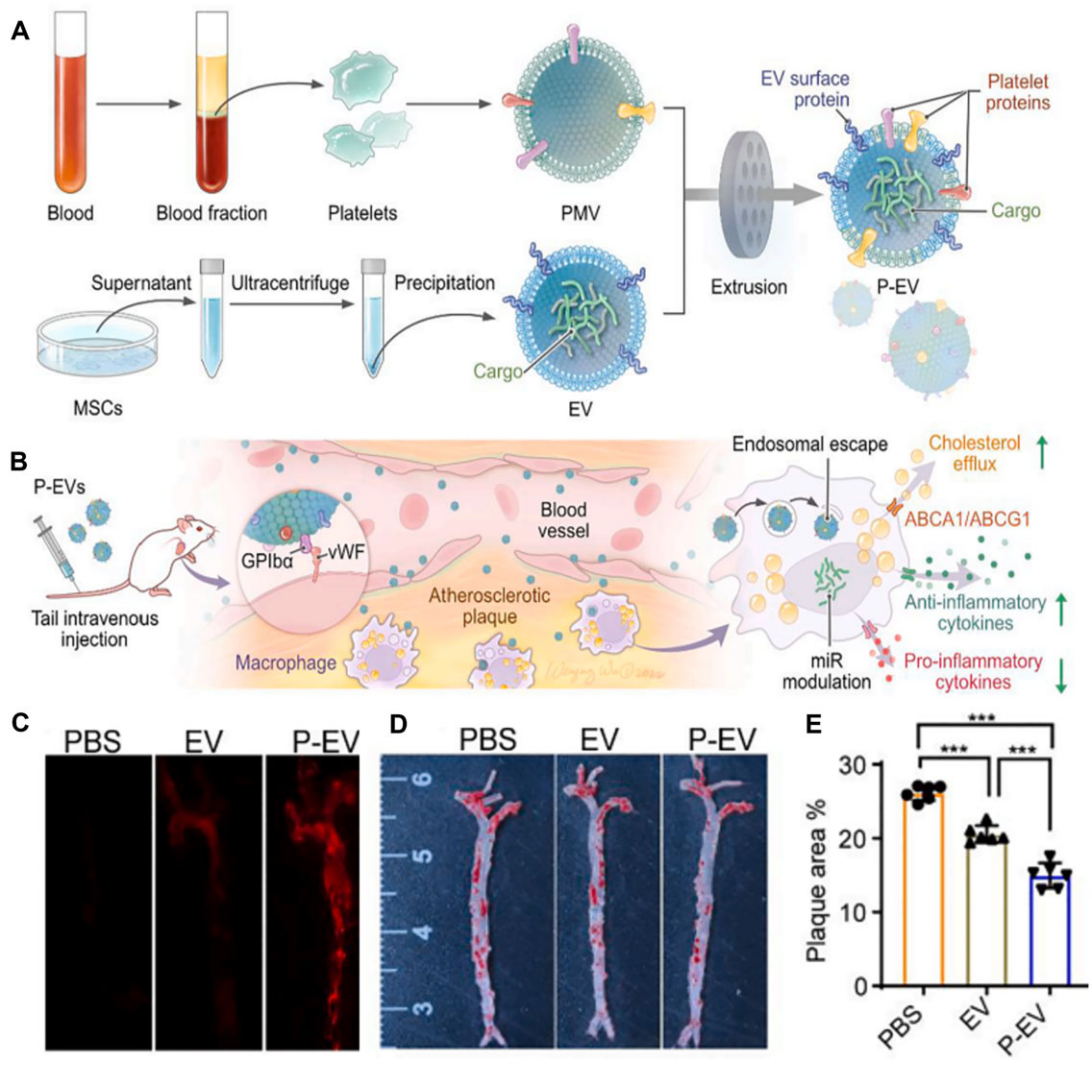
Targeted delivery of P-EVs for treatment of AS. (**A**) Preparation diagram of P-EV by fusing platelet membrane and MSC-EV and (**B**) treatment diagram of P-EV for treatment of AS. (**C**) Fluorescence images of the aortas after intravenous injection. (**D**) Images of the aortas after the treatment with different formulations and (**E**) quantitative data of plaque area in the aortas. Adapted with permission from Ref. [84].

Recently, mesenchymal stem cells (MSCs) have been repeatedly shown to have anti-AS effects [106, 107]. Li *et al.* fabricated the platelet membrane-modified MSCs-EVs (P-EVs) for targeting atherosclerotic plaque and regressing AS (Figure 7A and B) [84]. The P-EVs replicated the self-homing ability of platelets to the plaque and inherited the anti-inflammatory effect of MSCs-EVs. As shown in Figure 7C, the aortic fluorescence intensity was significantly stronger in the P-EV group than in the EVs group, indicating that the modification of the platelet membrane enhanced plaque targeting. The P-EVs significantly reduced the plaque area (14.96 ± 1.71%) compared with EVs (20.56 ± 1.16%) or PBS (26.15 ± 1.00%) group, exhibiting an excellent plaque reduction effect (Figure 7D and E).

In recent years, Exos have become promising nanocarriers to deliver proteins, nucleic acids and small-molecule drugs [103]. Bu *et al*. selected Exos-derived from HEK293T cells as nanocarriers to load and deliver inflammation-responsive IL-10 mRNA for AS anti-inflammation treatment [85]. Wu *et al.* molecularly engineered the anti-inflammatory M2 phenotype macrophages and obtained the engineered M2 Exos (HAL@M2 Exos) for drug-targeting inflammation delivery [86]. Compared to the free drug hexyl 5-aminolevulinate hydrochloride (HAL), HAL@M2 Exos could attain targeted delivery of HAL to plaques and effectively reduce inflammation for anti-AS. Bouchareychas *et al.* obtained Exos produced by naive bone marrow-derived M2 macrophage (BMDM-Exos) [108]. These BMDM-Exos contained miRNAs, such as miR-146b, miR-99a and miR-378a, which could transmit the anti-inflammatory properties of M2 macrophages, providing a new approach for AS treatment.

Studies indicated that exosomes derived from MSCs (MSCs-Exos) could play a vital role in tissue repair, immune regulation and disease treatment, providing a new strategy for clinical disease diagnosis and treatment [109]. Impaired efferocytosis function could hinder the clearance of apoptotic cells and necrotic cells in plaques, promoting the progression of AS [110, 111]. More importantly, the inability to remove apoptotic cells from atherosclerotic plaque under vascular stents could lead to the development of inflammation and the formation of severe occlusion. Moreover, these could severely impair the function of stents, leading to serious complications. Research indicated that MSCs could effectively enhance efferocytosis [109]. Zou *et al*. successfully extracted MSCs-Exos and encapsulated them with liposomes to construct multivesicular vesicles (MVVs) [112]. To this end, MVVs were grafted onto eluting vascular stent for AS treatment. Proteomic analysis and microRNA profiling showed that MSCs-Exos could regulate efferocytosis and accelerate apoptotic cell clearance. The MMV (MSCs-Exos) coating could inhibit thrombosis and prevent in-stent restenosis. In summary, exosomes derived from stem cells could be ideal drug carriers with promising therapeutic properties.

## Microenvironment-inspired nanocarriers

Many studies indicated that the vulnerable plaque microenvironment exhibited lower pH, higher ROS levels and richer lipids than normal tissue sites [2, 76, 113]. Considering the characteristics of the plaque microenvironment, the microenvironment-responsive nanocarriers have been designed and constructed to enable controlled drug release within atherosclerotic plaques (Table 2).

**Table 2.** Summary of nanocarriers inspired by the microenvironment

Biomimetic objects	Strategies	Nanocarriers	Functional moieties	Delivery effects	References
Microenvironment	pH-responsive	RBC@DTX/CR8	An acid cleavable ester bond	Controlled drug release in plaque	[76]
		Anti-miR33-loaded RAAM NP	α-CD and PEI	Effective nucleic acid drug delivery	[114]
	ROS-responsive	LFP/PCDPD	ROS-responsive polymer PMAEA	Controlled drug release in plaque	[115]
		Size-reducible HA-Fc/NP3ST	ROS-responsive ferrocene (Fc)	Deep plaque permeability	[116]
		NO-driven nanomotor PMA-TPP/PTX	Arginine (Arg)	Deep plaque permeability	[117]
		Carrier-free trehalose-based nanomotor Tr–Arg–PS	l-Arginine/phosphatidylserine (PS)	Deep plaque permeability/targeted macrophage delivery	[118]
	Cholesterol-responsive	Cargo-switching nanoparticles (CSNP)	CD	Controlled drug release in plaque	[119]

### pH-responsive nanocarriers

There have been some reports indicating that the atherosclerotic plaque presents a weak acidic (6.0–6.8) microenvironment [120], and the cellular endosomes/lysosomes show a strong acidic (4.5–5.5) microenvironment. These conditions provide an opportunity to design pH-responsive drug carriers to achieve drug release at the plaque site [121]. Zhong *et al*. developed a multifunctional platform with pH-sensitive prodrug and active targeting peptides [76]. The pH-sensitive prodrugs could remain stable and reduce drug leakage during circulation while being activated to release the drug in a controlled manner in response to the acidic conditions of atherosclerotic plaque. Li *et al*. designed a pH-responsive material based on acetylated α-cyclodextrin (AaCD) and polyethyleneimine (PEI) for the loading and delivering of microRNA-33 (miR-33) [114]. The pH-responsive anti-miR33 nanoparticles achieved desirable drug loading efficiency, high transfection and efficient endosomal/lysosomal escape, providing an efficient platform for AS treatment.

### ROS-responsive nanocarriers

**Figure 8.**
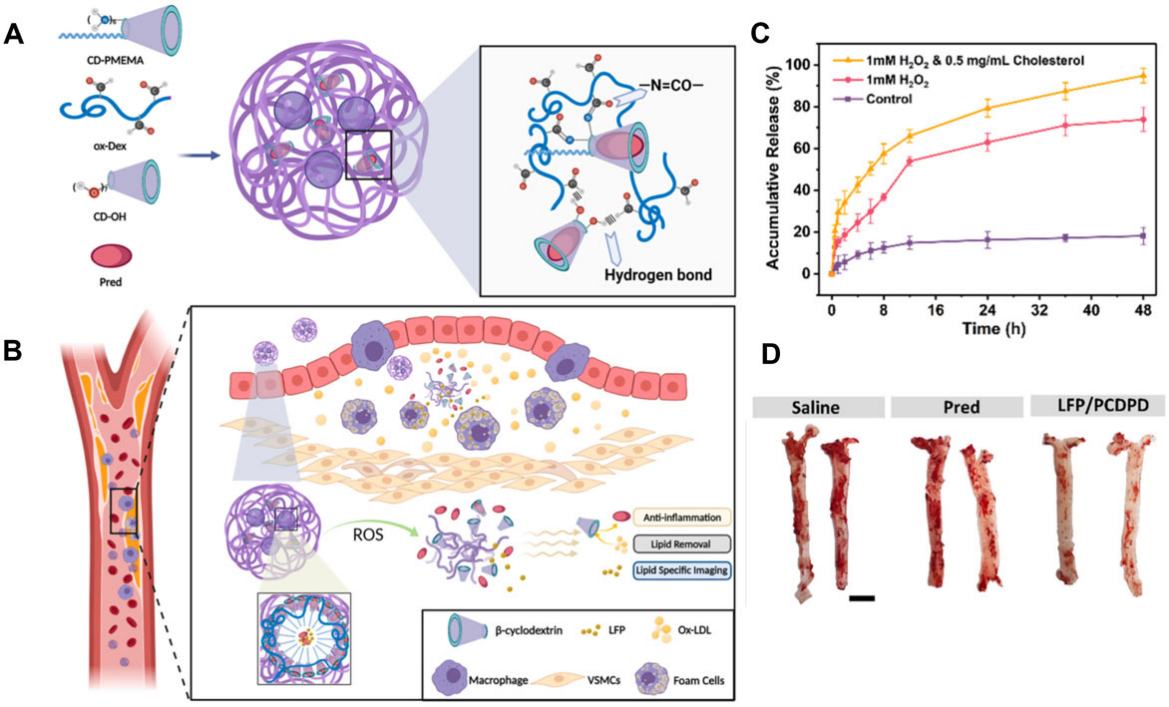
The dextran-based ROS-responsive nanoparticle, LFP/PCDPD, for targeted delivery and controlled release of drugs. (**A**) Preparation diagram and (**B**) treatment diagram of ROS triggered LFP/PCDPD. (**C**) Accumulative drug release of LFP/PCDPD under different solutions. (**D**) Photographs of the aortas treated with different formulations. Adapted with permission from Ref. [115].

Excess ROS in atherosclerotic plaque can be used as a precise trigger to achieve accurate and efficient drug release in atherosclerotic plaques [115, 122]. Zhao *et al.* synthesized a simvastatin prodrug by connecting PEG and simvastatin with an ROS-cleaved thioketal linker and designed an ROS-responsive nanoprodrug [122]. The nanoprodrug remained stable during circulation, and simvastatin was released at the plaque site due to the cleavage of thioketal linker in the presence of high ROS. Xu *et al.* selected the ROS-responsive material poly(2-(methylthio) ethyl methacrylate) (PMEMA) to construct stimulus-responsive LFP/PCDPD nanoparticles for the treatment of AS (Figure 8A and B) [115]. In detail, the hydrophobic PMEMA was attached to cyclodextrin to load the anti-inflammatory drug Prednisolone (Pred) and aggregation-induced emission fluorescent probe (LFP). As shown in Figure 8C, LFP/PCDPD nanoparticles showed an excellent stability in the control microenvironment without H_2_O_2_, with only minor 18.2% drug leakage occurring within 48 h (Figure 8C). In contrast, 75% drug was released after 48 h with over-expressed ROS, which was attributed to PMEMA changed from hydrophobic to hydrophilic, promoting structural disintegration and Pred release. Moreover, the lipid-rich microenvironment enhanced the aggregation of LFP within the plaque. The LFP/PCDPD with good ROS-responsive effect exhibited considerable anti-AS effort (Figure 8D).

**Figure 9.**
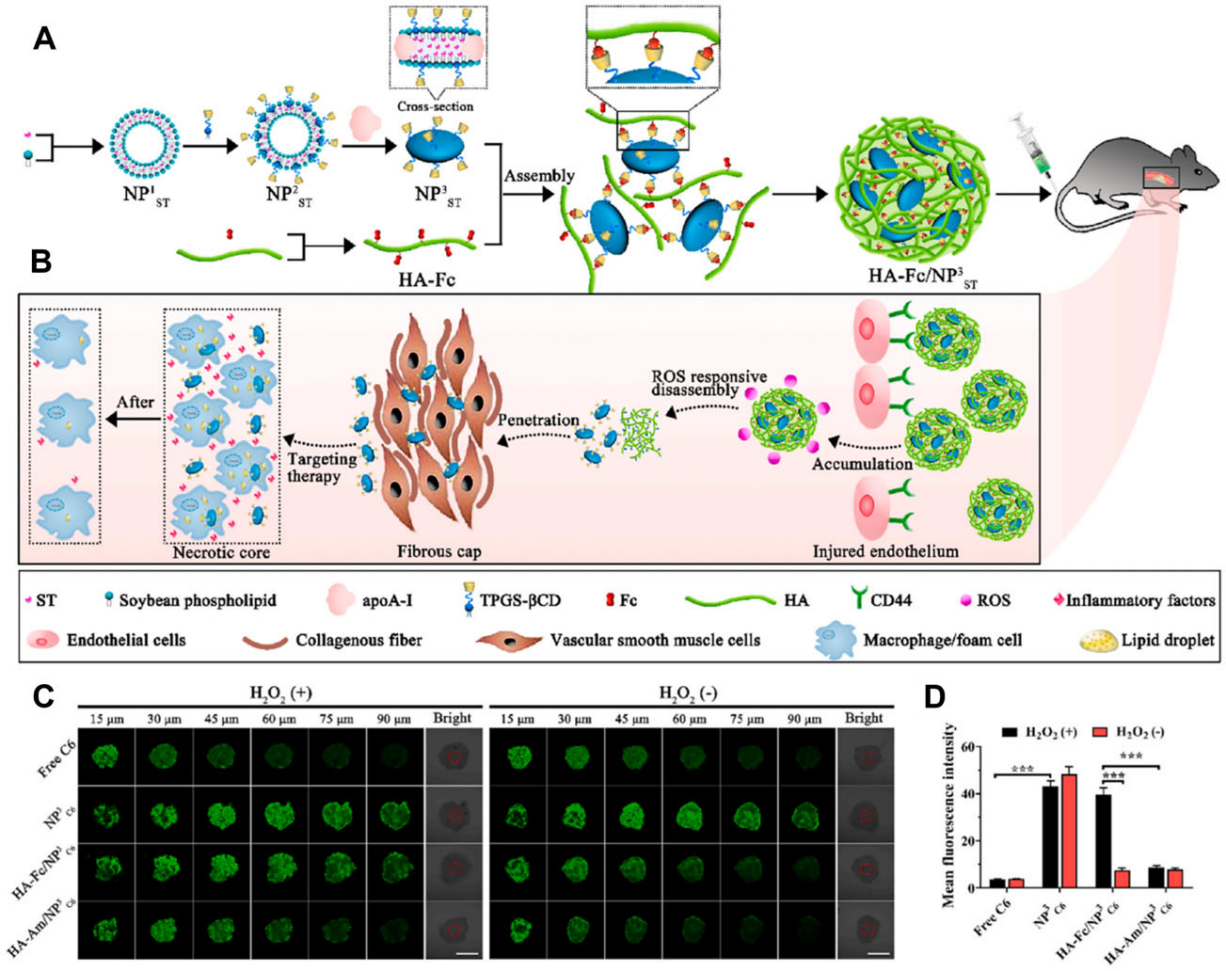
ROS-Responsive size-reducible HA-Fc/${\text{NP}}_{\text{ST}}^{3}$ nanoassemblies for targeted treatment of AS. (**A**) Preparation diagram and (**B**) treatment diagram of ROS-responsive HA-Fc/${\text{NP}}_{\text{ST}}^{3}$ nanoassemblies. (**C**) Penetration of different formulations in spheroids and (**D**) quantification of fluorescence intensity in the center of spheroids. Adapted with permission from Ref. [116].

In addition to acting as the trigger for the precise release of drugs, ROS can be used to improve the retention and penetration of drugs at plaque sites [116]. To reach the pathological macrophages/foam cells in atherosclerotic plaques, nanocarriers must pass through a thick fibrous cap composed of vascular smooth muscle cells and collagen fibers [123]. The size, shape and surface charge of nanoparticles determines their biological distribution in the body [124, 125]. In general, nanoparticles with a size of 100–200 nm possess a certain degree of blood circulation capacity, escaping the filtration by the liver and spleen. However, they suffer from certain difficulties in terms of penetrating atherosclerotic plaques [126]. Studies indicated that nanoparticles smaller than 30 nm could effectively penetrate tumors or plaques but were easily cleared during blood circulation [116, 126, 127]. Therefore, it is a feasible strategy to regulate the size of nanoparticles in response to the microenvironment, achieving both prolonged circulation and enhanced permeability. He *et al.* developed ROS-responsive size-reducible nanoassemblies based on host–guest interactions between β-CD and ferrocene (Fc) [116]. The simvastatin-loaded drug core (${\text{NP}}_{\text{ST}}^{3}$) was constructed using β-CD-anchored discoidal recombinant high-density lipoprotein. The cores were crosslinked by hyaluronic acid HA-Fc conjugates to form ROS-responsive nanoassemblies (HA-Fc/${\text{NP}}_{\text{ST}}^{3}$) (Figure 9A). The HA-Fc/${\text{NP}}_{\text{ST}}^{3}$ maintained a size of 100–200 nm during blood circulation, preventing them from being rapidly cleared. Then, the nanoassemblies targeted the injured endothelial cells due to the interaction of HA with the highly expressed CD44 receptors in the plaque. The hydrophobic Fc was converted into hydrophilic ferrocenium ions in the plaque microenvironment with excess ROS. This conversion promoted the decomposition between β-CD and Fc and the disassembly of the HA-Fc/${\text{NP}}_{\text{ST}}^{3}$. The released smaller ${\text{NP}}_{\text{ST}}^{3}$ further penetrated through the plaque and targeted macrophages to regulate cholesterol efflux, thus achieving high drug delivery efficiency and therapeutic effect (Figure 9B). As shown in Figure 9C and D, the HA-Fc/${\text{NP}}_{\text{ST}}^{3}$ exhibited stronger permeability in the presence of H_2_O_2_ compared to without H_2_O_2_. This enhanced permeability was attributed to the ROS-responsive nanoassemblies that released smaller drug cores. This work provided a new delivery strategy that utilized the ROS microenvironment to regulate the size of nanocarriers.

**Figure 10.**
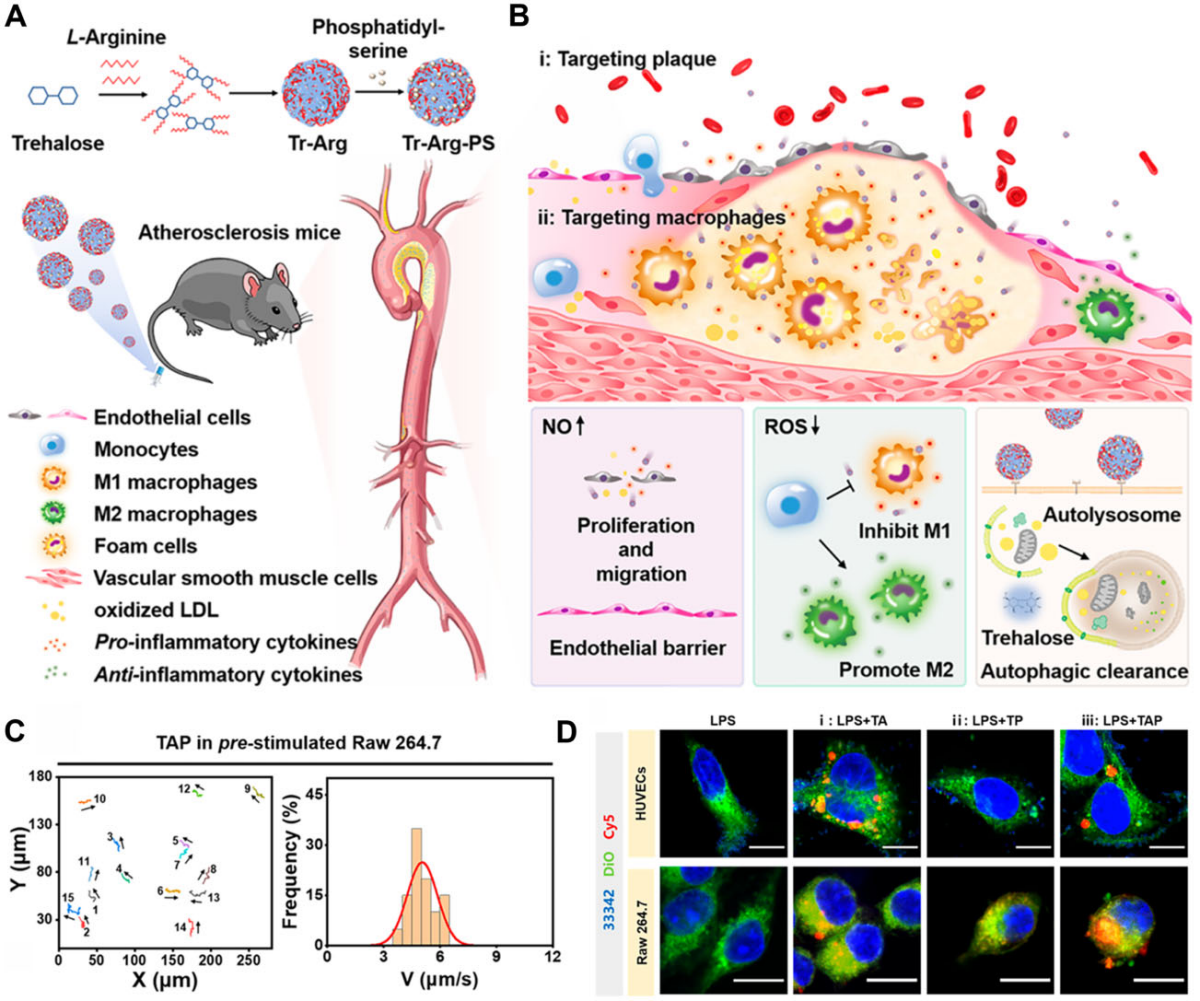
The carrier-free nanomotor TAP for targeting macrophages in vulnerable plaque. (**A**) The synthetic process of nanomotor TAP. (**B**) Treatment process diagram of TAP for targeting macrophages in vulnerable plaque. (**C**) The trajectory and the distribution of speed of TAP nanomotors. (**D**) Cellular uptake images of HUVECs and raw 264.7 treated with different formulations. Adapted with permission from Ref. [118].

The high level of ROS and the presence of inflammatory markers, such as inducible nitric oxide synthase (iNOS), in the plaque, can be used as chemical attractants to induce the chemotactic behavior of nanomotors [128]. Wu *et al.* constructed a carrier-free nanomotor, Tr–Arg–PS (TAP), for targeting macrophages in vulnerable plaque (Figure 10A and B) [118]. As shown in Figure 10C, the TAP nanomotors displayed an obvious motion trajectory in the Raw 264.7 cells, representing macrophages. The quantified fluorescence intensities of cell internalized TA and TAP nanomotors were 5.6 times and 3.6 times higher than for TP NPs (Figure 10D), which was attributed to the production of driving force nitric oxide (NO) between Arg and ROS in the plaque. The nanomotors were driven by to target plaque. Overall, the nanomotor technology provided a new drug delivery strategy for AS treatment.

### Cholesterol-responsive nanocarriers

**Figure 11.**
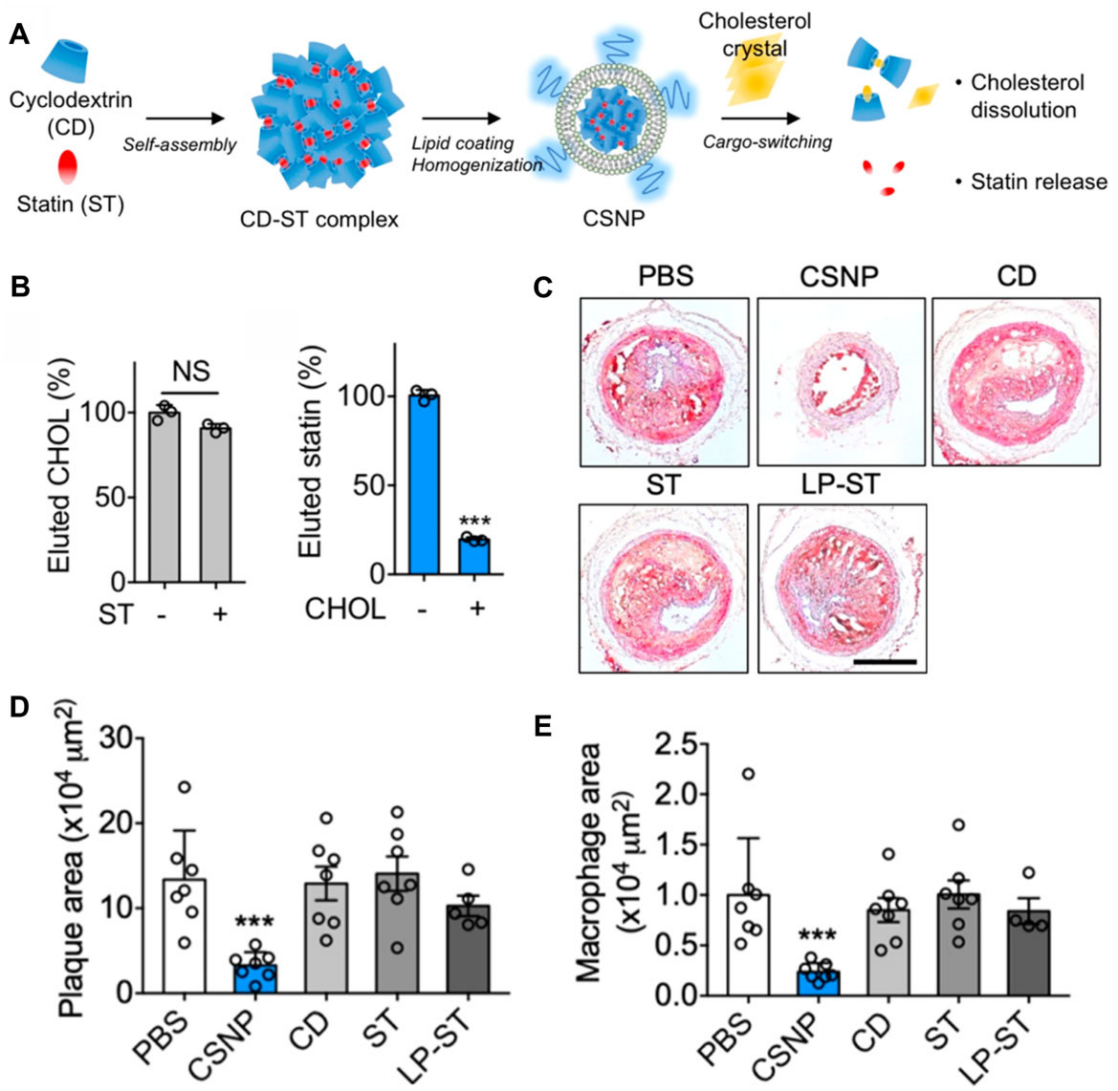
The CSNP for treatment of AS. (**A**) Schematic of CSNP preparation and cargo-switching. (**B**) Competitive binding of cholesterol (CHOL) and statin (ST) to CD. (**C**) Representative histological images of the left carotid artery (LCA) sections and (**D**) quantification of plaque area. (**E**) Quantification of macrophage area in the LCA sections. Adapted with permission from Ref. [119].

Cholesterol is the main component of atherosclerotic plaque [129]. Kim *et al*. constructed a cargo-switching nanoparticle (CSNP) driven by the affinity that used the cholesterol-rich microenvironment [119]. The core of CSNP, a cyclodextrin–statin (CD–ST) complex, was self-assembled by the affinity between β-CD and simvastatin (Figure 11A). Subsequently, phospholipids were coated on the core to form CSNP with core–shell structure. Further, a competitive binding assay was performed to test whether CD had a higher adsorption capacity for cholesterol or statins. As shown in Figure 11B, the elution of cholesterol was not significantly changed in the presence or absence of statin, whereas statin was markedly eluted in the presence of cholesterol. These results indicated that the β-CD showed a higher affinity to cholesterol than statin, enabling cholesterol-sensitive release of statin while depleting cholesterol. The treatment with CSNP significantly inhibited plaque growth and macrophage recruitment (Figure 11C–E).

## Summary and prospect

Nature-inspired nanocarriers, which take advantage of the natural properties of natural cells or the microenvironment, offer promising strategies for AS treatment. Combining synthetic functional materials and natural components, including lipids, proteins and membrane structures, can meet drug delivery needs [2, 47]. The functionality of nature-inspired nanocarriers predominantly stems from the physiological characteristics of source cells and the properties of synthetic materials. RBC membrane is the most typical example for constructing nanocarriers with prolonged blood circulation [87]. Other cells, such as platelets, white blood cells and macrophages, also have extended blood circulation property due to functional proteins on their surface [34]. Moreover, the development of atherosclerotic plaque is accompanied by the migration of various cells, such as platelets, white blood cells and macrophages [12, 130].

Consequently, these cells have also been used for targeted delivery [46, 79]. The adhesive atherosclerotic plaque constitutes a multifaceted microenvironment characterized by diverse cell types, rich cholesterol, inflammation and excess ROS [4]. Although the complex microenvironment limits drug delivery, microenvironment-responsive materials can be designed to achieve efficient drug delivery and controlled release [54, 115]. In summary, nature-inspired nanomedicines can meet four delivery needs: (i) avoid rapid blood clearance to achieve long blood circulation, (ii) targeted plaque delivery, (iii) targeted specific cell delivery, and (iv) controlled drug release at the action site.

While nature-inspired nanocarriers have demonstrated excellent potential for drug delivery, their fabrication, and effectiveness still impose limitations on their clinical progress [131]. Firstly, the natural membranes with complex lipids and proteins probably cause some unwanted reactions, posing challenges to safety *in vivo*. Synthetic materials, including the clinical material PEG, have specific immunogenicity [39]. Therefore, it is necessary to identify the composition of both natural and synthetic raw materials used in constructing nanocarriers. Secondly, the source of the natural materials determines their application. Currently, natural materials used in experiments are usually derived from cell lines or animals, which means that the application of nanocarriers is limited to the laboratory stage [46]. In such cases, the utilization of natural materials from the donor can significantly improve safety and effectiveness [131]. Thirdly, the production process of nanocarriers is not yet systematical. Extracting natural materials and preparing synthetic materials require optimization. The assembling strategies and storage duration need improvement. Moreover, the industry standards and manufacturing specifications are essential for the clinical application of nanomedicines. Despite these challenges for clinical application, the development of nature-inspired nanocarriers will provide novel and effective ideas for the treatment of AS.
